# Identification of bread wheat genotypes with superior grain yield and agronomic traits through evaluation under rust epiphytotic conditions in Kenya

**DOI:** 10.1038/s41598-021-00785-7

**Published:** 2021-11-01

**Authors:** Elizabeth Akinyi Msundi, James Otieno Owuoche, Maurice Edwards Oyoo, Godwin Macharia, Ravi Prakash Singh, Mandeep Singh Randhawa

**Affiliations:** 1 Department of Crops, Horticulture and Soils, Egerton University, P.O. Box 536-20115, Njoro, Kenya; 2 Food Crops Research Center, Kenya Agricultural and Livestock Research Organization (KALRO), Njoro, Kenya; 3 International Maize and Wheat Improvement Center (CIMMYT), Carretera México-Veracruz Km. 45, El Batan, 56237 Texcoco, Estado de México Mexico; 4 CIMMYT, World Agroforestry Center (ICRAF Campus), United Nations Avenue, PO Box 1041-00621, Gigiri, Nairobi Kenya

**Keywords:** Genetics, Plant sciences

## Abstract

Bread wheat (*Triticum aestivum* L.) cultivars adapted to specific environments and resistant to prevalent pathogens are preferred for obtaining high yield. This study aimed to identify wheat genotypes with superior grain yield (GY) and yield associated traits from 168 genotypes of International Maize and Wheat Improvement Center’s 13th Stem Rust Resistance Screening Nursery evaluated over two seasons during 2019 and 2020 under high disease pressure of both stem rust (SR) and yellow rust (YR) in a 21 × 8 *α*-lattice design with 3 replications in Kenya. Effects due to seasons were significant for YR_*Aud*_, SR_*Aud*_, 1000-kernel weight (TKW), days to heading (DH), plant height (PH) and number of spikelets spike^−1^ (SS), while genotypes and genotypes × season interaction effects were significant for all traits except number of kernels spike^−1^. Respectively, heritability values of 0.95, 0.93, 0.87, 0.86, 0.77 and 0.75 were observed for area under disease progress curve for SR (SR_*Aud*_), YR (YR_*Aud*_), TKW, DH, biomass (BM) and GY. Path analysis showed positive direct effects on GY via PH, SS, BM, and TKW. Biplot analysis identified 16 genotypes with superior desirable traits GY, BM and harvest index. The SR contributed the highest reduction in GY and TKW while YR contributed the most reduction in BM. These identified genotypes with superior GY combined with adequate resistance to both SR and YR are potentially valuable resources for improvement of locally adapted wheat cultivars.

## Introduction

Wheat (*Triticum aestivum* L.) is an important cereal food crop worldwide and it accounts for 21% of the global food demand with more than 80% of the global population depending on it as a source of protein and calories^1^. Globally, over 760 mt of wheat is produced annually from 220 mha of estimated area^2^. As global human population increases, wheat demand by 2050 is projected to increase by 33% at an annual rate of 1.6%^3^. The high demand aggregated by increasing population, prosperity, and a shift in dietary preferences to wheat products necessitates the need for ~ 40% increase in mean global wheat yield^4^. In Kenya, wheat is the second most important cereal food crop after maize, mainly cultivated in the Rift Valley with an average annual production of approximately 200,000 t from a cultivated area of 150,000 ha, with large-scale producers accounting for 80% of the production^5^. Kenya imports wheat to cover the deficit of over 60% to meet the annual demand of about 2 mt^6^.

Wheat production in Kenya is mainly affected by biotic stresses such as Russian wheat aphids (*Diuraphis noxia*), stem rust (*Puccinia graminis* f. sp*. tritici*, SR), yellow rust (*Puccinia striiformis* f. sp*. tritici*, YR) and Septoria tritici blotch diseases in most of the wheat growing areas^7,8^. Abiotic stresses such as drought, pre-harvest sprouting, and edaphic factors prevail in some areas and seasons^9^. In wheat, resistance or tolerance to biotic and abiotic stresses is partly controlled by a genotypic component. This provides an opportunity to develop adaptive and resilient wheat cultivars with higher yield^10^. Wheat breeding program at International Maize and Wheat Improvement Center (CIMMYT) has successfully incorporated the concept of diversity for disease resistance combined with agronomic traits resulting in high yielding cultivars adapted to diverse environments^11,12^. A superb example is the CIMMYT line *Attila* with a unique combination of Quantitative Trait Loci (QTLs)/genes for yield (*QYld*.*dms-2D*.*2*), test weight (*QTwt*.*dms-5A* and *QTwt*.*dms-5B*.*3*), four QTLs for kernel weight (*QTkw*.*dms-4A*, *QTkw*.*dms-6A*.*1*, *QTkw*.*dms-6D*.*2* and *QTkw*.*dms-7B*.*1*), adaptability to poor environments and resistance to both SR and YR which upon release became a popular cultivar in South Asia, Middle East, North Africa and East Africa^10,13^. However, *Atilla* and its derivative cultivars were shown to lack durable rust resistance when they succumbed to SR race Ug99 that evolved in East Africa^14^ and became susceptible to YR with the emergence of *Yr27*-virulent races in South Asia^15,16^. This demonstrated that success in achieving durable resistance combined with high yield relies on continued and consistent efforts to pyramid genes for these traits in new wheat cultivars^12^. Disease resistance has been shown to be highly correlated with high yield and quality of wheat^17^. For instance, Martin et al.^18^ described that leaf rust (*Puccinia triticina* Eriks) resistant lines with *Lr41* and *Lr42* increased grain yield by 63% and 26%, test weight by 5% and 3%, and kernel weight by 14% and 9%, respectively under high disease pressures.

Wheat is adapted to areas that experience a diurnal temperature of 3–4 °C minimum and 30–32 °C maximum (optimum of about 25 °C) and annual precipitation of 250–1750 mm (optimum range of 375–875 mm)^19^. Unfortunately, these optima for both temperature and moisture are highly conducive for the infection of rust diseases^20^. Thus, biotic stresses become main factors that contribute to the yield gap that exists between yield realized in experimental field where abiotic stresses are often controlled and those attained in farmers’ field^21,22^. Raising and/or maintaining wheat yield to their potential in farmers’ fields through conventional practices in absence of resistant cultivars, would require use of high rates of fungicides and fertilizers which can increase production costs and adversely affect the ecosystem^22,23^. This necessitates development of sustainable high yielding cropping practices^22^.

Yield is considered a quantitative trait as it is controlled by minor effect genes. It is largely influenced by environmental factors which create the need to estimate both genotypic and environmental effects on yield^24^. Correlations (*r*), heritability estimates (*H*^2^) and path coefficient statistics have been used to study phenotypic variations in wheat germplasm for both agronomic and disease resistance traits^25,26^. For instance, Morgounov et al*.*^27^ noted a negative association of severe SR infections in CIMMYT breeding lines and cultivars with both kernel size and yield. Bhatta et al.^25^ used broad sense heritability estimates and principal component biplot analyses to identify 5 lines that combined high yield, better quality, and multiple disease resistance from 143 hexaploid synthetic and bread wheat in Western Siberia. For interpretation of biplots, angles between vectors and inner products are inferred using trigonometric laws of cosines that relates to length of sides of a triangle to the cosine of one of its angles^28^. The approximated correlation coefficients (*r*) between any 2 given traits is determined by the cosine of angle between their vectors, where *r* = cos 90° = 0 (no correlation), *r* = cos 180° = − 1 (negative correlation) and *r* = cos 0° = 1 (positive correlations)^28,29^. Improvement in efficiency for yield selection is likely if the pathways by which yield is reached can be classified. Path analysis^26^ has been used to estimate direct and indirect effects of yield components on yield of synthetic near isogenic CIMMYT wheat lines evaluated in Texas in presence of LR and SR infections^30^. Given the importance of breeding for high yielding and rust resistant wheat cultivars, particularly in Kenya, where the most devastating SR and YR races prevail, this study was conducted to identify genotypes with superior grain yield and yield associated traits relative to commercialized Kenyan cultivars under rust epiphytotic conditions.

## Results

### Temperature and rain fall

Respectively, mean minimum temperature of 9.6 °C and 10.6 °C and maximum temperature of 22.8 °C and 23.8 °C were noted during main season 2019 (MS2019) and off season 2020 (OS2020). Mean monthly rainfall of 657.5 mm was recorded during MS2019 and 681.9 mm during OS2020. The summary of the mean temperature and rainfall observed during both seasons is given in Table 1.

**Table 1:** Mean temperature (minimum and maximum), total rainfall, mean monthly rainfall and total number of rainy days recorded during seasons MS2019 and OS2020 at Njoro.

Season	Mean temperature (°C)		Total rainfall (mm)	Mean monthly rainfall (mm) ± SE	Number of rainy days
	Min ± SE	Max ± SE			
MS2019	9.6 ± 0.39	22.8 ± 0.37	657.5	105.7 ± 20.00	69
OS2020	10.6 ± 0.39	23.8 ± 0.39	681.9	120.6 ± 39.11	61

*SE* standard error.

### Analysis of variance and genotypes’ performance across seasons

Effects due to seasons were significant area under disease progress curve for stem rust (SR_*Aud*_) and yellow rust (YR_*Aud*_) at *p * < 0.001, 1000-kernel weight (TKW), grain yield (GY), days to heading (DH), plant height (PH) and number of spikelets spike^−1^ (SS) at *p * < 0.05, however, they were not significant (*p * > 0.05) for spike length (SL), number of kernels spike^−1^ (KS) and biomass (BM). Effects due to both genotypes and genotype × season interaction were significant for SR_*Aud*_ and YR_*Aud*_ (*p * < 0.01). Genotype effects were significant (*p * < 0.01) for all yield-related traits except for KS whereas season × genotype interaction was significant (*p * < 0.001) for all yield-related traits (Table 2).

**Table 2:** Mean squares of the wheat genotypes evaluated for stem rust (SR), yellow rust (YR), grain yield (GY) and yield related traits during MS2019 and OS2020 at KALRO, Njoro.

Source of variation	df	Expected mean squares	YR_*Aud*_	SR_*Aud*_	TKW	GY	SS	DH
Season	1	δ^2^_ε_ + 336 δ^2^_b_ + 7056 δ^2^_r_ + 21,168 δ^2^_s_	561,944.44**	1,825,888.69**	27,575.79*	275.79*	126.93*	1800.01*
Replicates/season	4	δ^2^_ε_ + 336 δ^2^_b_ + 7056 δ^2^_r_	9833.77***	52,005.60***	28.53***	24.62*	6.60**	102.79***
Blocks/replicates × season	120	δ^2^_ε_ + 336 δ^2^_b_	1230.63	7333.30*	2.57	0.60*	1.43	4.99
Genotypes	167	δ^2^_ε_ + 126 δ^2^_sg_ + 256 δ^2^_g_	45,963.70***	653,376.30***	82.67***	7.74***	3.56*	126.40***
Season × genotypes	167	δ^2^_ε_ + 126 δ^2^_sg_	3417.56***	34,905.20***	10.49***	1.88***	2.72***	17.70***
Error	548	δ^2^_ε_	1161.60	5600.20	2.17	0.48	1.30	4.79
CV (%)			30.96	22.98	6.48	17.69	6.32	2.94
*R*^2^			0.94	0.98	0.94	0.90	0.68	0.92

Source of variation	df	Expected mean squares	PH	SL	KS	BM	HI	
Season	1	δ^2^_ε_ + 336 δ^2^_b_ + 7056 δ^2^_r_ + 21,168 δ^2^_s_	1148.59*	0.78	61.85	9274.36	0.02	
Replicates/season	4	δ^2^_ε_ + 336 δ^2^_b_ + 7056 δ^2^_r_	54.48*	10.80***	632.84***	2405.04***	0.07***	
Blocks/replicates × season	120	δ^2^_ε_ + 336 δ^2^_b_	17.37	0.31	56.06*	28.98	0.00	
Genotypes	167	δ^2^_ε_ + 126 δ^2^_sg_ + 256 δ^2^_g_	56.32***	1.60***	85.81	234.96***	0.01***	
Season × genotypes	167	δ^2^_ε_ + 126 δ^2^_sg_	31.97***	0.58***	73.81***	59.25***	0.00***	
Error	548	δ^2^_ε_	14.75	0.25	43.42	23.20	0.00	
CV (%)			4.33	5.29	14.45	16.36	20.21	
*R*^2^			0.72	0.78	0.61	0.86	0.83	

*YR*_*Aud*_ yellow rust area under disease progress curve (AUDPC), *SR*_*Aud*_ stem rust AUDPC, *TKW* thousand kernel weight, *GY* grain yield, *SS* number of spikelets spike^−1^, *DH* days to 50% heading, *PH* plant height, *SL* spike length, *KS* number of kernels spike^−1^, *BM* biomass, *HI* harvest index, *CV* coefficient of variation, *R*^*2*^ coefficient of determination. The expected mean squares determined the random error as a test for the blocks and the genotype × season effects, the replicates as an error term to test the effects due to seasons, the genotype × season as an error term to test the effect due to genotypes and blocks as an error term to test the effect due to replicates; ***, ** and * = significance at *p* < 0.001, *p* < 0.01 and *p* < 0.05, respectively.

During MS2019, SR infection resulted in 23.12% higher mean SR_*Aud*_ than observed in OS2020. However, low YR infection was observed during MS2019 with YR_*Aud*_ means that were 35.32% less than those of OS2020. Generally, wheat lines in MS2019 performed better agronomically with plants that were 3.53% earlier in heading and 2.38% shorter than performance in OS2020. The MS2019 also produced lines with higher SS, GY, TKW, mean kernel weight (MKW) and BM exceeding the OS2020 season by 3.86%, 39.53%, 8.06%, 8.70% and 18.70%, respectively (Table 3).

**Table 3:** Mean of SR_Aud_, YR_Aud_, grain yield and yield related traits for the genotypes evaluated during MS2019 and OS2020 at KALRO, Njoro.

Season	YR_*Aud*_	SR_*Aud*_	DH	PH	SL	SS	KS	BM	GY	TKW	MKW	HI
				cm				t ha^−1^		g		
MS2019	86.49b	368.22a	72.96b	87.71b	9.56a	18.37a	45.86a	32.47a	5.59a	23.70a	0.023a	0.14a
OS2020	133.71a	283.10b	75.63a	89.85a	9.50a	17.66b	45.36a	26.40b	3.38b	21.79b	0.021b	0.13b
MSD	4.21	9.26	0.27	0.48	0.06	0.14	0.82	0.60	0.17	0.18	0.0004	0.003

Means followed by the same letters are not significantly different at *p * < 0.05.

*YR*_*Aud*_ yellow rust area under disease progress curve (AUDPC), *SR*_*Aud*_ stem rust AUDPC, *TKW* thousand kernel weight, *GY* grain yield, *SS* number of spikelets spike^−1^, *DH* days to 50% heading, *PH* plant height, *SL* spike length, *KS* number of kernels spike^−1^, *BM* biomass, *HI* harvest index, *MSD* Tukey’s minimum significance difference.

Overall, genotypes produced GY that ranged from 1.16 to 8.98 t ha^−1^ with a mean GY of 4.48 t ha^−1^, while SR_*Aud*_ ranged from 60.08 to 1825.83 and YR_*Aud*_ ranged from 2.92 to 472.50 (Table 4). Although 80% of lines produced heavier kernels than those from checks, 45% of lines produced superior GY. About 10% of lines including 6015, 6026, 6045, 6046, 6051, 6069, 6070, 6071, 6104, 6110, 6114, 6120, 6133, 6161, 6163 and 6168 produced GY greater than 6 t ha^−1^. Among these, high yielding lines, 6069, 6110, 6120, 6163, 6168, 6071, 6046, 6104 and 6114 displayed SR_*Aud*_ ranging from 0.0 to 300.0 whereas YR_*Aud*_ ranged from 0.0 to 150.0. The TKW ranged from 10.81 to 31.80 g with heaviest kernels from lines 6070, 6046 and 6016 (Table 5).

**Table 4:** Mean and range of SR_*Aud*_, YR_*Aud*_, grain yield and yield related traits of wheat genotypes and checks evaluated during MS2019 and OS2020 at KALRO Njoro.

Variables	Range	Mean	SE	Resistant check		Susceptible checks					
				Kingbird		Robin		PBW343		Cacuke	
				Mean	Rank	Mean	Rank	Mean	Rank	Mean	Rank
YR_*Aud*_	2.92–472.50	110.10	7.25	85.75	83	79.92	77	215.85	149	321.08	161
SR_*Aud*_	60.08–1825.83	325.66	27.19	112.00	19	1825.83	168	1615.00	161	1767.500	165
DH	65.83–87.83	74.29	0.39	72.50	73	75.17	102	70.50	43	66.67	3
PH (cm)	76.40–97.60	88.78	0.27	84.97	24	93.53	152	88.50	84	82.47	3
SL (cm)	8.41–10.97	9.53	0.04	8.70	159	9.50	87	9.54	77	10.21	19
SS	15.17–20.18	18.02	0.06	17.30	141	19.17	16	18.27	61	17.30	141
KS	36.66–60.09	45.61	0.30	51.59	9	45.88	77	44.05	109	39.79	158
TKW (g)	10.81–31.80	22.75	0.31	19.71	135	12.46	165	13.65	163	15.00	160
MKW (g)	0.01–0.03	0.02	0.04	0.02	135	0.01	166	0.01	163	0.02	160
GY (t ha^−1^)	1.16–8.98	4.48	0.11	3.70	128	1.32	166	1.16	168	1.51	163
BM (t ha^−1^)	11.97–47.92	29.43	0.53	22.38	145	20.83	152	12.92	167	14.23	165
HI	0.06–0.27	0.15	0.03	0.17	71	0.06	168	0.09	162	0.11	153

*YR*_*Aud*_ yellow rust area under disease progress curve (AUDPC), *SR*_*Aud*_ stem rust AUDPC, TKW-thousand kernel weight, *GY* grain yield, *SS* number of spikelets spike^−1^, *DH* days to 50% heading, *PH* plant height, *SL* spike length, *KS* number of kernels spike^−1^, *BM* biomass, *HI* harvest index, *SE* standard error, Rank-performance of the checks when ranked from the best to the worst performing genotype (n = 168). DH and PH was ranked from the earliest to the latest and from the shortest to the tallest, respectively, SL, SS, KS, TKW, MKW, GY and BM were ranked from the highest to the lowest while YR_*Aud*_ and YR_*Aud*_ were ranked from the least value (most resistant) to the highest value (most susceptible).

**Table 5:** Performance of the 16 high yielding genotypes evaluated for grain yield, 1000-kernel weight, and SR and YR resistance.

Genotype number	GID	Pedigree	Resistance type or known gene^a^	GY (t ha^−1^)	TKW (g)	SR_*Aud*_	YR_*Aud*_
6069	8,044,987	ALTAR84/AE.SQUARROSA(221)//3*BORL95/3/URES/JUN//KAUZ/4/WBLL1/5/MUTUS/6/SUP152/BAJ #1	APR^b^	8.98	29.91	95.08	106.75
6110	8,048,083	SAUAL/3/ACHTAR*3//KANZ/KS8584/4/SAUAL/5/2*BAJ#1/3/KIRITATI//ATTILA*2/PASTOR	APR	8.69	29.08	241.50	2.92
6120	8,051,474	KACHU/DANPHE//KENYA SUNBIRD/KACHU	*SrND643* + APR	8.35	25.81	303.92	119.58
6163	8,043,600	FRNCLN//KIRITATI/2*TRCH/3/FRNCLN/4/SWSR22T.B./2*BLOUK#1//WBLL1*2/KURUKU	*Sr22* + APR	7.82	29.38	76.42	2.92
6168	8,043,914	KENYASUNBIRD/KACHU*2//BORL14	*SrND643* + APR	7.38	28.01	246.17	100.33
6071	8,044,993	ALTAR 84/AE.SQUARROSA (221)//3*BORL95/3/URES/JUN//KAUZ/4/WBLL1/5/MUTUS/6/SUP152/BAJ #1	APR	7.22	29.92	102.08	74.67
6046	8,050,160	KANCHAN*2/JUCHI//2*BOR L14	APR	7.19	30.69	218.17	8.75
6104	8,048,389	KACHU#1//WBLL1*2/KUKUNA/3/BRBT1*2/KIRITATI/6/ROLF07*2/5/REH/HARE//2*BCN/3/CROC1/AE.SQUARROSA(213)//PGO/4/HUITES/7/BORL14	APR	7.10	26.41	182.00	56.00
6114	8,059,265	MEX94.27.1.20/3/SOKOLL//ATTILA/3*BCN/4/2*SUP152*2/TECUE #1	APR	7.02	29.37	181.42	2.92
6070	8,044,989	ALTAR 84/AE.SQUARROSA (221)//3*BORL95/3/URES/ JUN//KAUZ/4/WBLL1/5/MUTUS/6/SUP152/BAJ #1	APR	6.98	31.80	120.75	49.00
6026	8,049,385	SLVS/ATTILA//WBLL1*2/3/GONDO/CBRD/4/BORL14	APR	6.96	29.12	300.42	67.67
6161	8,044,291	KACHU*2/3/ND643//2*PRL/2*PASTOR/4/KACHU/DANPHE	APR	6.90	24.29	160.42	82.25
6015	8,049,046	SERI.1B*2/3/KAUZ*2/BOW//KAUZ/4/FRANCOLIN #1/5/ MUNAL/6/KACHU #1/KIRITATI//KACHU	APR	6.54	28.89	179.67	120.17
6133	8,052,137	WBLL1*2/BRAMBLING*2//BAVIS/3/MISR 1	*Sr13a* + APR	6.39	23.53	60.08	39.08
6051	8,050,806	SUP152/VILLA JUAREZ F2009/3/2*ATTILA*2/PBW65//MURGA	APR	6.29	26.13	147.58	163.92
6045	8,050,123	PRL/2*PASTOR*2//FH6-1-7/3/2*ATTILA*2/PBW65//MURGA	APR	6.09	26.95	107.92	137.08
Kingbird	4,799,764	TAM-200/TUI/6/PAVON-76//CAR-422/ANAHUAC-75/5/BOBWHITE/CROW//BUCKBUCK/PAVON-76/3/YECORA-70/4/TRAP-1	APR	3.70	19.71	112.00	85.75
Cacuke	5,347,441	CANADIAN/CUNNINGHAN//KENNEDY	Susceptible check	1.51	15.00	1767.5	321.08

*GID* CIMMYT’s genotype identification number, *YR*_*Aud*_ yellow rust area under disease progress curve (AUDPC), *SR*_*Aud*_ stem rust AUDPC, *TKW* thousand kernel weight, *GY* grain yield.

^a^Unpublished data (Mandeep Randhawa, CIMMYT, Kenya).

^b^Adult Plant Resistance.

### Variance components and broad sense heritability

Genetic variance (*σ*_*G*_) for all traits except PH, KS, SL and SS surpassed variance for season (*σ*_*E*_), genotype × season (*σ*_*G*×*E*_) and error (*σ*_*e*_) though the genetic, season, genotype × season and error variance for MKW and HI were negligible. The highest proportion of genetic variance relative to environmental variance was exhibited by SR_*Aud*_ (82.23%), YR_*Aud*_ (61.47%), TKW (47.13%) and DH (34.15%) while environmental variance was higher than the genotypic variance for number of KS (96.45%), SS (90.16%), PH (75.21%), SL (48.72%), BM (41.23%) and GY (33.93). The highest broad sense heritability (*H*^2^) was observed for SR_*Aud*_ (0.95) and YR_*Aud*_ (0.93)*.* The TKW, MKW, DH, BM and GY showed *H*^2^ greater than 0.75 while *H*^2^ of 0.60 and 0.67 was exhibited by HI and SL, respectively. Low *H*^2^ was detected for KS (0.13) and SS (0.29) (Table 6).

**Table 6:** Estimates of genotypic variance (*σ*^2^_*G*_), environment (season) variance (*σ*^2^_*E*_), genotype × environment (season) variance (*σ*^2^_*G*×*E*_), error variance (*σ*^2^_*e*_) and broad sense heritability (*H*^2^) for YR_*Aud*_, SR_*Aud*_, yield and yield related traits of evaluated genotypes.

Variable	*σ*^2^_*G*_	*σ*^2^_*E*_	*σ*^2^_*G*×*E*_	*σ*^2^_*e*_	*H*^2^
PH	5.89 ± 1.44	2.21 ± 3.22	6.57 ± 1.30	14.98 ± 0.83	0.50
GY	1.11 ± 0.17	0.54 ± 0.77	0.52 ± 0.08	0.62 ± 0.03	0.75
MKW	0.00 ± 0.00	0.00 ± 0.00	0.00 ± 0.00	0.00 ± 0.00	0.82
HI	0.00 ± 0.00	0.00 ± 0.00	0.00 ± 0.00	0.00 ± 0.00	0.60
KS	1.90 ± 2.16	0.03 ± 0.26	11.42 ± 2.91	42.48 ± 2.36	0.13
SL	0.20 ± 0.04	0.00 ± 0.00	0.11 ± 0.02	0.28 ± 0.02	0.67
SS	0.18 ± 0.09	0.27 ± 0.39	0.55 ± 0.10	1.01 ± 0.06	0.29
TKW	13.75 ± 1.74	1.81 ± 2.59	3.22 ± 0.44	2.24 ± 0.12	0.87
DH	21.73 ± 2.81	3.53 ± 5.05	5.37 ± 0.79	5.40 ± 0.30	0.86
BM	34.64 ± 5.09	18.65 ± 26.56	11.14 ± 2.35	29.15 ± 1.62	0.77
YR_*Aud*_	8205.00 ± 972.00	1107.00 ± 1577.00	901.00 ± 142.00	1153.00 ± 64.00	0.93
SR_*Aud*_	116,937.00 ± 13,548.00	3544.00 ± 4123.00	11,236.00 ± 1453.00	6000.00 ± 333.00	0.95

*PH* plant height, *GY* grain yield, *MKW* mean kernel weight, *HI* harvest index**,** *KS* number of kernels spike^−1^, *SL* spike length, *SS* number of spikelets per spike, *TKW* thousand kernel weight, *DH* days to 50% heading, *BM* biomass, *YR*_*Aud*_ yellow rust area under disease progress curve (AUDPC), *SR*_*Aud*_ stem rust AUDPC.

### Correlation and path analyses

Path analysis revealed positive direct effects on GY of 39.50% via KS (0.14), 63.47% via TKW (0.50) and 65.98% via BM (0.45). Although the direct effects on GY via SS (0.02) were positive, a negative association (nearly negligible) was observed between GY and SS (*r* = − 0.03). Negative direct effects on GY were observed via DH (− 0.13), PH (− 0.03) and SL (− 0.07). Although the correlation between GY and DH (*r* = − 0.30) and between GY and SL (*r* = − 0.16) were equally negative, the association between GY and PH (*r* = 0.14) was positive (Table 7).

**Table 7:** Direct (diagonal) and indirect effects of the yield related traits on grain yield of genotypes evaluated during seasons MS2019 and OS2020 at KALRO Njoro.

Trait	DH	PH	SL	SS	KS	TKW	BM	Correlation with GY	VIF
DH	− 0.13258	**− **0.00747	**− **0.00324	0.00071	**− **0.01940	**− **0.19573	0.05403	**− **0.30368***	1.40523
PH	**− **0.03695	− 0.02680	**− **0.01881	0.00346	0.03214	**− **0.04506	0.23455	0.14256	1.87812
SL	**− **0.00617	**− **0.00724	− 0.06956	0.00650	0.03448	**− **0.10440	**− **0.01415	**− **0.16052*	1.27615
SS	**− **0.00411	**− **0.00405	**− **0.01974	0.02293	0.02615	**− **0.03448	**− **0.01370	**− **0.02707	1.12639
KS	0.01901	**− **0.00637	**− **0.01774	0.00443	0.13534	0.09019	0.11776	0.34263***	1.25284
TKW	0.05165	0.00240	0.01446	**− **0.00157	0.02428	0.50249	0.19805	0.79174***	1.93316
BM	**− **0.01606	**− **0.01409	0.00221	**− **0.00071	0.03571	0.22304	0.44619	0.67629***	2.31553

*DH* days to 50% heading, *PH* plant height, *SL* spike length, *SS* number of spikelets spike^−1^, *KS* number of kernels spike^−1^, *TKW* thousand kernel weight, *BM* biomass**,** *GY* grain yield, *VIF* variance inflation factor; ***, ** and * = significance at *p* < 0.001, *p* < 0.01 and *p* < 0.05, respectively.

### Genotype × Trait biplot and trait relationship analyses

**Figure 1:**
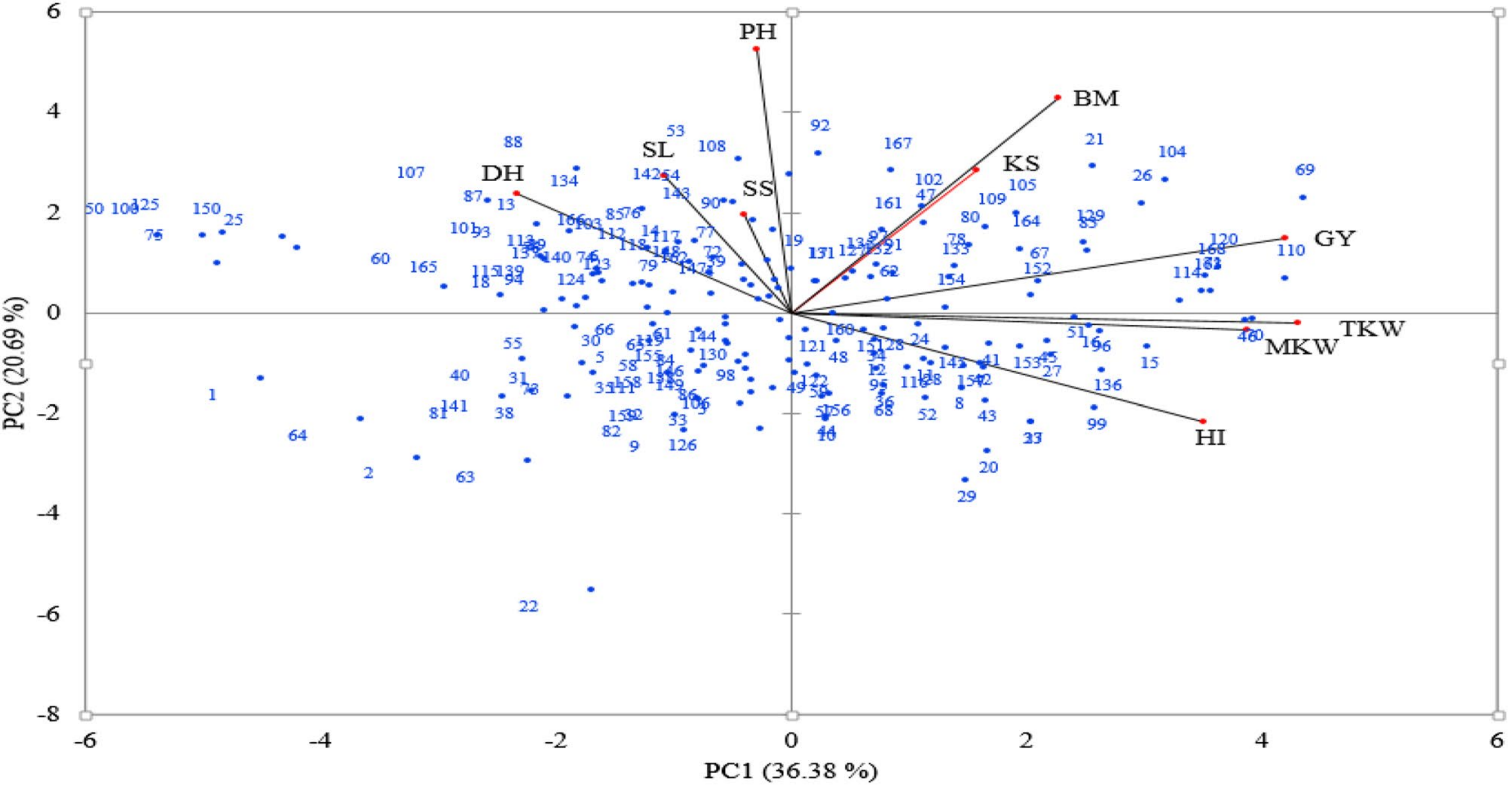
Which-won-where biplot of the 168 genotypes evaluated for grain yield and yield related traits on the first 2 principal components. Here, *TKW* thousand kernel weight, *GY* grain yield, *SS* number of spikelets spike^−1^, *DH* days to 50% heading, *PH* plant height, *SL* spike length, *KS* number of kernels spike^−1^, *BM* biomass, *HI* harvest index. Blue labels represent genotype numbers from 1 to 168 for respective entries from 6001 to 6168. The black and red lines represent trait projections on 2 axes, greater the projection, higher the contribution to total variations in a given axis.

GY as well as yield related traits were plotted on principal component (PC) axes to depict the proportion of variance contributed by each trait. Of the 10 axes of differentiation, only the first 3 PCs showed Eigen values > 1 and cumulatively accounted for 71.20% of total variation. The first PC explained 36.38% of the total variation with the highest contribution from TKW (factor loading = 0.93) followed by GY (factor loading = 0.91) and MKW (loading = 0.84) while the second and third PCs accounted for 20.69% and 14.13% of the variation, respectively. Biplot of the yield related traits on the first and second PC showed acute angles among BM, KS, GY, TKW and MKW which depicted positive correlations among these parameters. Acute angles were also observed among DH, SS, SL and PH. It was revealed that high yielding lines 6069, 6104, 6120, 6168, 6163, 6070, 6160, 6026, 6110, 6051, 6114, 6016, 6083, 6021 and 6129 showed high values for TKW, MKW, KS and BM whereas the low yielding genotypes were tall and late with long spikes and more SS (Fig. 1).

### Simple and stepwise regression analysis

A simple regression of GY on YR_*Aud*_ resulted in a model that explained 13% of the variations in a model with a slope of − 0.005 while a similar regression on YR_*Aud*_ yielded a model with *R*^2^ of 0.22 with a slope of − 0.002. Response of TKW due to YR_*Aud*_ showed a *β-*coefficient of − 0.008 with only 4% variations in the model explained while SR_*Aud*_ resulted in a *β*-coefficient of − 0.006 with 33% of variation explained by the fitted model. The analysis revealed higher contribution to biomass reduction by YR_*Aud*_ with an *R*^2^ of 0.44 compared to 0.05 for SR_*Aud*_*.* Stepwise regression analysis revealed that SR_*Aud*_ played significant role in influencing the TKW with model *R*^2^ of 0.33 and *C*_*p*_ statistic of 18.29. However, a partial value of *R*^2^ = 0.06 that resulted into model *R*^2^ of 0.39 was observed for a model with both SR_*Aud*_ and YR_*Aud*_ with a *C*_*p*_ value of 3.00. GY was negatively influenced by SR_*Aud*_ with model and partial *R*^2^ of 0.22 and *C*_*p*_ of 42.33. However, YR_*Aud*_ was the second variable with a model *R*^2^ of 0.38 and *C*_*p*_ of 3.00. Lastly, contrary to GY and TKW, the results showed that YR_*Aud*_ contributed to the reduction of BM with *R*^2^ of 0.44 and *C*_*p*_ statistic of 28.51 while a model with both SR_*Aud*_ and YR_*Aud*_ resulted in a partial *R*^2^ of 0.08, model *R*^2^ of 0.52 and a *C*_*p*_ of 3.00 (Table 8).

**Table 8:** Regression coefficients for SR_*Aud*_, YR_*Aud*_, 1000-kernel weight, biomass, and yield of the test genotypes.

Regressand	Regressor	Regression equation	*R*^2^		
**Simple regression**					
GY	YR_*Aud*_	GY = 5.077 − 0.005 YR_*Aud*_	0.13		
	SR_*Aud*_	GY = 5.095 − 0.002 SR_*Aud*_	0.22		
TKW	YR_*Aud*_	TKW = 23.713 − 0.008 YR_*Aud*_	0.04		
	SR_*Aud*_	TKW = 24.850 − 0.006 SR_*Aud*_	0.33		
BM	YR_*Aud*_	BM = 34.751 − 0.048 YR_*Aud*_	0.44		
	SR_*Aud*_	BM = 30.892 − 0.004 SR_*Aud*_	0.05		

			Model *R*^2^	Partial *R*^2^	*C*_*p*_
**Step wise regression**					
GY	SR_*Aud*_	GY = 5.095 − 0.002 SR_*Aud*_	0.22	0.22	42.33
	SR_*Aud*_, YR_*Aud*_	GY = 5.789 − 0.002 SR_*Aud*_ − 0.006 YR_*Aud*_	0.38	0.16	3.00
TKW	SR_*Aud*_	TKW = 24.850 − 0.006 SR_*Aud*_	0.33	0.33	18.29
	SR_*Aud*_, YR_*Aud*_	TKW = 26.093 − 0.007 SR_*Aud*_ − 0.011 YR_*Aud*_	0.39	0.06	3.00
BM	YR_*Aud*_	BM = 34.751 − 0.048 YR_*Aud*_	0.44	0.44	28.51
	SR_*Aud*_, YR_*Aud*_	BM = 36.701 − 0.005 SR_*Aud*_ − 0.049 YR_*Aud*_	0.52	0.08	3.00

*GY* grain yield, *TKW* thousand kernel weight, *BM* biomass, *SR*_*Aud*_ stem rust area under disease progress curve (AUDPC), *YR*_*Aud*_ yellow rust AUDPC, *R*^*2*^ coefficient of determination, *Partial* *R*^*2*^ coefficient of partial determination-indicates the proportion of variation explained by the full model that could not be explained by the regressor in the reduced model, *Model R*^*2*^ adjusted coefficient of determination-the best model is the one with the largest model *R*^*2*^, *C*_*p*_ Mallows *C*_*p*_ statistic: indicates amount of bias in estimating the regression coefficient and therefore predicting the response.

## Discussion

In the present study, significant effects due to seasons indicated that seasonal conditions that prevailed in MS2019 were entirely different from OS2020 and this influenced the performance of wheat genotypes over growing seasons in different years. The mean monthly rainfall and temperature was higher in OS2020 than in MS2019, thus creating conducive relative humidity for rust infection that resulted in yield reduction. Differential performance among different CIMMYT’s High Rainfall Wheat Yield Nurseries for yield across low and high rainfall seasons have been reported^31^.

Effects due to genotypes were significantly different for various traits like DH, PH, SL, SS, TKW, GY, BM, HI, SR_*Aud*_ and YR_*Aud*_ indicating presence of high level of genetic diversity among genotypes hence genotypes with desirable traits can be selected for use in local breeding. Based on the findings from this study, the MS2019 could be suitable for selection of genotypes for traits including earliness, shortness, higher SS, GY, TKW, MKW and BM since despite higher SR infections the lines had higher means for these traits compared to OS2020. Of the evaluated genotypes, 51 had multiple sibs sharing common parents in different genetic backgrounds. Phenotypic variation of these lines for above said traits suggests these lines were subjected to selection procedures across diverse environments. These variations could have been combined through the shuttle breeding strategy adopted by CIMMYT through which early generation segregating populations are evaluated in contrasting environments to identify appropriate genetic variation for wide adaptation, durable rust resistance and to enhance yield gains^32^.

Significant genotype × season interaction observed for studied traits indicated differential response of genotypes to environments resulting in non-uniform phenotypic response of lines to SR, YR, yield and yield-related traits. This variation could be either due to interaction of the genetic and non-genetic factors during plant growth or possibly presence of diverse genes or their combination in these genotypes with differential efficiency to control growth and response to rust infections. Previous studies reported significant genotype × environment interactions for yield and yield components in wheat genotype^33,34^. Therefore, plant breeders should take climatic factors into consideration when selecting genotypes that are stable across environments to avoid abandoning good breeding lines and/or carrying over poor genetic stocks.

The high proportion of genetic variance relative to environmental variance exhibited by SR_*Aud*_, YR_*Aud*_, TKW, PH, DH and GY indicates the expression of these traits was under minimal environmental influence. On the other hand, higher estimates of environmental variance relative to genotypic variance for KS, SS, PH, SL and BM suggested that genotypes exhibited variable response for these traits across the seasons with greater influence by environmental conditions, therefore, selection based on phenotypic value of observed traits is unreliable. Knowledge of heritability is appropriate for predicting response to selection of a particular trait under certain environmental conditions. Moreover, it helps determine whether or not a particular trait can be improved by selection, by improvement of management practices, or both. Theoretically, genotypes with broad genetic background selected in contrasting environments would be expected to have low broad sense heritability for target traits due to occurrence of high G × E interaction variance which results in unreliable ranking of genotypes across environments^35^. High heritability observed for both SR_*Aud*_ and YR_*Aud*_, indicates a large proportion of observed variance is heritable and selection for these traits is potentially effective, although this is dependent on the magnitude of dominance and epistatic effects which constitutes a proportion of genetic variance that is non heritable. Comparatively higher heritability of 83.09% for GY were observed in F_3_ segregating populations evaluated in Pakistan^36^.

A positive correlation of GY was observed with PH, KS, TKW and BM, however, the direct effects of PH on GY were negative. On the other hand, negative correlation of GY with DH, SS and SL were observed but the direct effects of SS on GY were positive indicating that the undesirable effect of SS on GY was influenced by other traits. Other studies have reported correlation and path analysis on agronomic traits in wheat^37,38^. Although late maturity is normally associated with more accumulation of dry matter which translates into high GY, the negative relationship between earliness and GY observed in this study is desirable as an escape strategy in case heat and drought stresses prevail during growth. Despite the fact that PH had a positive though not significant correlation with GY, its direct effects on yield were negative which could imply that the indirect effects of other traits on PH significantly impacted yield. The high direct effects on GY via KS, TKW and BM indicate that these traits can be used indirectly as a selection criterion to improve GY.

The biplot analysis enabled a visual comparison of the traits, genotypes, and their interrelationships. It displayed the patterns of variability of the traits on the first and second principal components as accounting for 57.07% of the total variations present. This proportion can be considered low, with a reflection on the complexity of the relationships among the evaluated traits, in accordance with the findings of comparatively lower variability of 68% in a study by Mohammadi et al.^39^ who evaluated Iranian durum wheat varieties for plant height, grain yield, days to maturity and a 1000-kernel weight across rain fed and irrigated environments. In a related study, Bhatta et al.^25^ noted 54% total variation on the first 2 principal components associated with agronomic and quality traits of synthetic and bread wheat accessions in Western Siberia. Traits TKW, MKW, BM, PH and HI had longer vectors and were more responsive in discriminating between lines that performed well for these traits against the ones that relatively performed low. Positive correlation between TKW, MKW, BM, KS, GY and HI as indicated by acute angles in biplot suggested that genotypes plotted along these vectors potentially possess multiple desirable traits that can be selected simultaneously for development of high yielding genotypes with good agronomic attributes. Applying a genotype by trait biplot in a study on soybean (*Glycine max* L.) cultivars in Ontario, Yan and Rajcan^29^ were also able to visually compare and select promising cultivars for multiple traits including seed yield, oil content, protein content, plant height and days to maturity.

From regression analyses, reduction in GY and TKW was better explained by SR_*Aud*_ whereas YR_*Aud*_ better explained the reduction in BM. Effects of SR on GY in wheats evaluated across different environments in Kenya have been previously reported^40^. Stem rust normally infects stem sheaths, leaves and occasionally glumes of wheat plant, and both mesophyll and palisade layers are ruptured during rust establishment^41^. Estimated gains in dry weight of spring wheat kernel result from temporary accumulation of photosynthates in plant stems near the time of anthesis and if SR infection occurs around this critical stage, it is expected to exert a negative influence on GY^42^.

The reduction in yield and grain quality due to rust infection could be due to reduction of photosynthetic area and destruction of phloem tissue that are responsible for mobilization and remobilization of photosynthates. Severe infections due to compatible reaction between rust and host genotype result into altered phloem transport to divert nutrients to actively growing urediniospores at the expense of developing spikes of wheat resulting in shriveled kernels and hence poor yield^41^. Though YR does not destroy tissues as done by SR, it severely infects and kills leaves at vegetative and reproductive stages through destruction of photosynthetic functions of leaves and affecting movement of assimilates, consequently reduces biomass that is often observed at physiological maturity^43^. This phenomenon was clearly observed in the step wise regression analysis, since high YR infections contributed to a greater reduction of the BM compared to SR infection which contributed the largest reduction in GY and TKW.

This study demonstrates that small plot yield tests along with data on yield related traits, YR and SR are useful for preliminary screening of a large set of lines to identify promising lines for large scale yield testing. Additionally, small plot yield trials help in strategic use of resources in terms of field space, labor and time that are increasingly required to conduct large size yield plots for large number of entries. However, according to a study by Fischer and Kertesz^44^, small plot wheat yield and harvest index estimation, respectively, explained 46% and 53% of the variations that could occur in large plot testing. This scenario was also evident in this study where the harvest index, which is a predictor of the yielding ability of genotypes showed comparatively lower range than those reported in most experiments performed in large plot testing, which reflects on the influence of interplant and interplot competitions that occurs in small plots. We found that most of the wheat genotypes possessing adult plant resistance (APR) to both rusts performed better than genotypes carrying either a race specific resistance gene or combination of race specific resistance genes. We identified 16 genotypes that possess adequate level of resistance to both SR and YR, and superior GY and yield-related traits. These genotypes would serve as a valuable resource for the selection or further improvement of locally adapted wheat cultivars.

## Methods

### Plant material

A set of 168 wheat genotypes of CIMMYT’s 13th Stem Rust Resistance Screening Nursery (SRRSN) along with SR susceptible check cultivars (Cacuke, PBW343 and Robin) and resistant check cultivar (Kingbird) were evaluated. The 168 wheat genotypes are derived from diverse parents. One hundred and eleven genotypes were derived as single selections from specific crosses whereas 57 were sibs.

### Location

Field trials were conducted to evaluate agronomic and yield performance during the main-season 2019 (MS2019: June to October) and off-season 2020 (OS2020: January to May) at the Kenya Agricultural and Livestock Research Organization (KALRO), Njoro. This is located at 35° 55′ 60″ E, 0° 19′ 60″ S with an elevation of 2185 m above sea level in lower highland agro-ecological zone III (LH3)^45^ with predominant well-drained mollic andosols soils. The experimental site on the average has annual precipitation of about 1000 mm, with minimum and maximum temperature of 9 °C and 22 °C, respectively (KALRO Meteorological station No. 903502 (1), 2015).

### Field preparation, trial design and crop management

A well-drained plain field that was previously under a cover crop of canola (*Brassica napus*) was used for this study. Land was disc ploughed once and harrowed twice to achieve a fine tilled seedbed suitable for planting wheat. Each line was planted in a 2-row plot measuring 0.2 m × 0.75 m, separated by 0.2 m alley spacing between rows and 0.5 m spacing between adjacent blocks and replicates^5^ at an equivalent seeding rate of 125 kg ha^−1^ in a 21 × 8 *alpha* lattice design with 3 replicates. At sowing time, 22.5 kg N ha^−1^ and 25.1 kg P ha^−1^ were supplied from an application of diammonium phosphate (DAP) at an equivalent rate of 125 kg ha^−1^. A single row of a spreader mixture of SR susceptible wheat genotypes [Cacuke, Robin and six *Sr24*-carrying lines (CIMMYT GIDs: 5391050, 5391052, 5391056, 5391057, 5391059 and 5391061)] was planted perpendicular to the entries as disease spreaders within the replicates after every 2 blocks and as quad rows around the experimental unit. Artificial stem rust epidemic was created through syringe inoculation of disease spreader plants at growth stage (GS) 47^46^ with a suspension of urediniospores of SR races TTKSK, TTKTK, TTKST and TTKTT in distilled water mixed with 1 mg L^−1^ of Tween 20.

During both MS2019 and OS2020, the field was immediately irrigated using sprinklers after planting to supply adequate moisture to initiate germination and seedling growth. Supplemental irrigation was done when the rainfall was inadequate. After planting, a pre-emergence herbicide Stomp 455C was applied to supply *pendimethalin* at an equivalent rate of 1.37 kg ha^−1^ while *Buctril* MC a post emergence herbicide, was applied at GS13^46^ to supply *Bromoxynil octanoate* at an equivalent rate of 0.28 kg ha^−1^ + *MCPA ethyl-hexyl ester* at 0.28 kg ha^−1^, both mixed at a rate of 1.25 kg ha^−1^ to selectively control annual broad leaf weeds. Calcium ammonium nitrate (CAN) was top dressed when plants attained GS30^46^ at an equivalent rate of 100 kg ha^−1^ to supply 33 kg N ha^−1^. Systemic insecticide Thunder OD 145 was applied at a rate of 0.25 kg ha^−1^, to supply *imidacloprid* at 0.03 kg ha^−1^ and *beta-cyfluthrin* at 0.01 kg ha^−1^ at tillering (GS 25) and ear emergence (GS 55) stages^46^ to control Russian wheat aphid.

### Data collection

The first round of natural infections of YR disease severity evaluation was done when about 50% of the test genotypes headed and the susceptible checks showed 50% disease severity. Evaluation of genotypes for YR was done over at least 2 occasions. Later, genotypes were evaluated for SR when susceptible checks showed 50% SR severity and notes were taken over 3 occasions. In all instances, YR and SR severities were estimated at 7-day intervals based on the modified Cobb’s Scale (0–100%)^47^. Phenological traits viz. DH, PH, SL, KS, SS, BM, HI, TKW and MKW along with GY were measured for all wheat genotypes. For each genotype, plants were considered to have headed when 50% part of spike emerged from the boot. PH was measured at physiological maturity from the base of the plant at the soil level to the tip of the spikes excluding awns from a random sample of 5 plants per genotype. The SL was measured from a random sample of 5 spikes from base to tip excluding the awns. Both KS and SS were determined from a sample of 5 random spikes per plot. At physiological maturity, plots were harvested by cutting at the base for estimating GY and BM. Both GY and BM were recorded in grams per plot area (g m^−2^) then converted into t ha^−1^ as:

$$\text{GY }\left(\text{t }\;{\text{ha}}^{-1}\right)= \frac{{10, 000\;m}^{2}}{plot\;area \left({m}^{2}\right)}\times \frac{{GY \; PLOT}^{-1} \left(g\right)}{1,000, 000 \; g}\; \text{and \; BM }\left(\text{t }\;{\text{ha}}^{-1}\right)=\frac{{10, 000\;m}^{2}}{plot \; area \left({m}^{2}\right)}\times \frac{{BM \; PLOT}^{-1}\left(g\right)}{1000, 000 g}.$$

The HI was computed by determining the ratio of GY to the total BM of plants upon harvesting from samples obtained from each plot. One thousand kernels were counted from threshed grains using a Contador seed counter (brand *Pfeuffer*, Serial number: 14176107) and weighed to estimate TKW. The MKW was estimated by dividing the TKW by 1000 seeds for each genotype.

### Data analyses

Both SR and YR rust severity notes were converted into area under disease progress curve^48^. Combined analysis of variance (ANOVA) across seasons was performed using general linear model (GLM) procedure in SAS software^49^ (version 9.1.3) using below equation:

$$Y_{ijklm} = \, \mu + \, S_{i} + \, R_{j \, (i)} + \, B_{k \, (ij)} + \, G_{l} + \, SG_{il} + \varepsilon_{ijklm} ,$$

where *Y*_*ijklm*_ is the observation of experimental units, *µ* is the overall mean, *S*_*i*_ is the effect due to *i*th season, *R*_*j (i*)_ is the effect due to *j*th replicate in the *i*th season, *B*_*k (ij*)_ = effect due to *k*th block in the *j*th replicate in the *i*th season, *G*_*l*_ is the effect due to *l*th genotype in the *k*th block in the *j*th replicate, *SG*_*il*_ is the effect due to interaction between *i*th season and *l*th genotype in the *i*th season in the *j*th replicate and *ε*_*ijklm*_ is the random error component. Effects due to genotypes were considered fixed while replicates, blocks, season and genotype × season effects were treated as representatives hence considered random. To test all pairwise comparisons among means of seasons, the Tukey’s test was calculated for each trait whenever season effects were significant^50^.

Estimates of genetic, genotype × environment (genotype × season) and error variance components were computed using *PROC MIXED* procedure in SAS software using restricted maximum likelihood (REML) method with genotype as a random factor. These components were used to estimate broad sense heritability (*H*^2^) on genotype mean basis, given by:

$${H}^{2}=\frac{{\sigma }_{G}^{2}}{{\sigma }_{P}^{2}}=\frac{{\sigma }_{G}^{2}}{{\sigma }_{G}^{2}+\frac{{\sigma }_{GE}^{2}}{E}+\frac{{\sigma }_{e}^{2}}{ER}},$$

where *σ*^*2*^_*P*_ is the total phenotypic variance, *σ*^*2*^_*G*_ is the genotypic variance, *σ*^*2*^_*GE*_ is the genotype × year variance, *σ*^*2*^_*e*_ is the error, *R* is the number of replications and *E* is the number of seasons^51^.

Pearson correlation analysis was conducted using the *PROC CORR* procedure in SAS software to establish the relationship among GY and yield related traits. For Path analysis^26^, multicollinearity test was first checked among all independent variables using variance inflation factor statistic. To partition the correlation coefficients for yield related traits into direct and indirect effects contributing to GY, the *PROC CALIS* procedure of SAS^49^ was used. To depict the proportion of variance explained by each yield component and associations among parameters, a biplot of genotypes and trait analysis was performed in XLSTAT Microsoft excel-2013 add-in software^52^.

A regression analysis was performed to determine the contribution of SR and YR infections on the GY losses of rust infected wheat genotypes. This was conducted using the *PROC REG* forward selection method in SAS software where phenotypic value of a given trait was modeled as:

$${Y}_{i }={\beta }_{o }+{\beta }_{1}{X}_{1}+{\beta }_{2}{X}_{2}+{\varepsilon }_{i},$$

where *Y*_*i*_ is the expected value of dependent variable for a set of independent variables *X*_1_*,* and *X*_2_; *β*_0_ is the expected value of dependent variable at *X*_1_ or *X*_2_, = 0;* β*_1_,* β*_2_, is the partial regression coefficients for every unit increase or decrease in independent variables *X*_1_ and *X*_2_, respectively and ${\varepsilon }_{i}$ is the random error.

### Use of plant material

Experimental research and field studies on common wheat plants used in this study, including the collection of plant material, were conducted following relevant institutional, national, and international guidelines and legislation. Permission from Kenya Plant Health Inspectorate Service (KEPHIS) was obtained for import of wheat seeds in Kenya.

## Data Availability

The data used to present the findings reported in this study is available upon request through the corresponding author.

## References

[1] Shewry PR (2009). Wheat. J. Exp. Bot..

[2] Shiferaw B (2013). Crops that feed the world 10. Past successes and future challenges to the role played by wheat in global food security. Food Secur..

[3] FAO. The State of food insecurity in the world—Addressing food insecurity in protracted crises. *Food and Agriculture Organization of the United Nations, Rome, Italy* (2010). http://www.fao.org/3/i1683e/i1683e.pdf.

[4] Fischer, R. A., Byerlee, D. & Edmeades, G. *Crop Yields and Global Food Security: Will Yield Increase Continue to Feed the World? ACIAR Monograph no. 158*. (Australian Centre for International Agricultural Research, 2014).

[5] Kamwaga, J. *et al*. *Kenya Wheat Production Handbook*, vol. 78. (Kenya Agricultural and Livestock Research Organization, 2016).

[6] Gitau, R., Mburu, S., Mathenge, M. K. & Smale, M. *Trade and Agricultural Competitiveness for Growth, Food Security and Poverty Reduction: A Case of Wheat and Rice Production in Kenya*. Working Papers 202596. (Egerton University, Tegemeo Institute of Agricultural Policy and Development, 2011). 10.22004/ag.econ.202596.

[7] Njau, P. *et al.* Combating stem rust to protect wheat crops in Kenya. In *Proc. Borlaug Global Rust Initiative 2009 Technical Workshop*, 17–20 March (Ciudad Obregon, 2009).

[8] Njuguna MN, Mwangi MM, Kamundia JK, Koros I, Ngotho G (2016). Cultural management of Russian wheat aphid infestation of bread wheat varieties in Kenya. Afr. Crop Sci. J..

[9] Macauley, H. & Ramadjita, T. Cereal crops: Rice, maize, millet, sorghum, wheat. In *Feeding Africa*, October 21–23, Dakar, Senegal, 7 (2015).

[10] Rajaram, S. & Braun, H. J. Wheat yield potential. In *Proc*. *International Symposium on Wheat Yield Potential: Challenges to International Wheat Breeding*. 103–107 (International Maize and Wheat Improvement Center (CIMMYT), 2008).

[11] Rajaram, S., Borlaug, N. E. & Van Ginkel, M. CIMMYT international wheat breeding. In *Bread Wheat: Improvement and Production*. 103–117 (Food and Agriculture Organization of the United Nations (FAO), 2002).

[12] Singh RP (2014). Progress towards genetics and breeding for minor genes based resistance to Ug99 and other rusts in CIMMYT high-yielding spring wheat. J. Integr. Agric..

[13] Zou J (2017). QTLs associated with agronomic traits in the Attila × CDC Go spring wheat population evaluated under conventional management. PLoS One.

[14] Pretorius ZA, Singh RP, Wagoire WW, Payne TS (2000). Detection of virulence to wheat stem rust resistance gene Sr31 in *Puccinia graminis* f. sp. *tritici* in Uganda. Plant Dis..

[15] Prashar M, Bhardwaj SC, Jain SK, Datta D (2007). Pathotypic evolution in *Puccinia striiformis* in India during 1995–2004. Aust. J. Agric. Res..

[16] Solh, M., Nazari, K., Tadesse, W. & Wellings, C. R. The growing threat of stripe rust worldwide. In *Proc. Borlaug Global Rust Initiative (BGRI) 2012 Technical Workshop*, September 1–4, Beijing, China (2012).

[17] Singh RP (2016). Disease impact on wheat yield potential and prospects of genetic control. Annu. Rev. Phytopathol..

[18] Martin JN, Carver BF, Hunger RM, Cox TS (2003). Contributions of leaf rust resistance and awns to agronomic and grain quality performance in winter wheat. Crop Sci..

[19] Sivakumar MVK, Valentin C (1997). Agro-ecological zones and the assessment of crop production potential. Philos. Trans. R. Soc. Lond. Ser. B Biol. Sci..

[20] Leonard KJ, Szabo LJ (2005). Stem rust of small grains and grasses caused by *Puccinia graminis*. Mol. Plant Pathol..

[21] Curtis BC, Rajaram S, Gómez Macpherson H (2002). Bread Wheat: Improvement and Production.

[22] Licker R (2010). Mind the gap: How do climate and agricultural management explain the ‘yield gap’ of croplands around the world?. Glob. Ecol. Biogeogr..

[23] Gaba S, Gabriel E, Chadœuf J, Bonneu F, Bretagnolle V (2016). Herbicides do not ensure for higher wheat yield, but eliminate rare plant species. Sci. Rep..

[24] Ahmad F (2011). Genetic analysis of some quantitative traits in bread wheat across environments. Afr. J. Agric. Res..

[25] Bhatta M (2019). Marker-trait associations for enhancing agronomic performance, disease resistance, and grain quality in synthetic and bread wheat accessions in Western Siberia. G3 Genes Genomes Genet..

[26] Wright SEWALL (1983). On, “Path analysis in genetic epidemiology: A critique”. Am. J. Hum. Gen..

[27] Morgounov A (2020). Genetic basis of spring wheat resistance to leaf rust (*Puccinia triticina*) in Kazakhstan and Russia. Euphytica.

[28] Kroonenberg PM (2008). Applied Multiway Data Analysis. International Statistical Review.

[29] Yan W, Rajcan I (2002). Biplot analysis of test sites and trait relations of soybean in Ontario. Crop Sci..

[30] Cooper JK (2012). Increasing hard winter wheat yield potential via synthetic wheat: I. Path-coefficient analysis of yield and its components. Crop Sci..

[31] Gerard GS (2020). Grain yield genetic gains and changes in physiological related traits for CIMMYT’s High Rainfall Wheat Screening Nursery tested across international environments. Field Crops Res..

[32] Ortiz R (2007). High yield potential, shuttle breeding, genetic diversity, and a new international wheat improvement strategy. Euphytica.

[33] Akçura M, Kaya Y, Taner S (2005). Genotype-environment interaction and phenotypic stability analysis for grain yield of durum wheat in the Central Anatolian Region. Turk. J. Agric. For..

[34] Aycicek M, Yildirim T (2006). Path coefficient analysis of yield and yield components in bread wheat (*Triticum aestivum* L.) genotypes. Pak. J. Bot..

[35] Mangi SA (2010). Heritability studies for grain yield and yield components in F3 segregating generation of spring wheat. Pak. J. Bot..

[36] Sial MA, Arain MA, Ahmad M (2000). Genotype × environment interaction on bread wheat grown over multiple sites and years in Pakistan. Pak. J. Bot..

[37] Tsegaye D, Dessalegn T, Dessalegn Y, Share G (2012). Genetic variability, correlation and path analysis in durum wheat germplasm (*Triticum durum* Desf). Agric. Res. Rev..

[38] Gelalcha S, Hanchinal RR (2013). Correlation and path analysis in yield and yield components in spring bread wheat (*Triticum aestivum* L.) genotypes under irrigated condition in Southern India. Afr. J. Agric. Res..

[39] Mohammadi M, Sharifi P, Karimizadeh R, Shefazadeh MK (2012). Relationships between grain yield and yield components in bread wheat under different water availability (dryland and supplemental irrigation conditions). Notulae Botanicae Horti Agrobotanici Cluj-Napoca..

[40] Wanyera R (2016). Management of wheat rusts at different growth stages using Nativo 300 SC (*trifloxystrobin* 100g/L+ *tebuconazole* 200g/L) fungicide. Aust. J. Crop Sci..

[41] Roelfs, A. P., Singh, R. P. & Saari E. E. Rust diseases of wheat: Concepts and methods of disease management. Mexico, D.F., CIMMYT 1-81 (1992).

[42] Romig RW, Calpouzos L (1970). The relationship between stem rust and loss in yield of spring wheat. Phytopathology.

[43] He C (2019). Study on stripe rust (*Puccinia striiformis*) effect on grain filling and seed morphology building of special winter wheat germplasm Huixianhong. PLoS One.

[44] Fischer RA, Kertesz Z (1976). Harvest index in spaced populations and grain weight in microplots as indicators of yielding ability in spring wheat. Crop Sci..

[45] Jaetzold, R., Schmidt, H., Farm Management Handbook of Kenya. Volume II B. Central Kenya. Ministry of Agriculture, Kenya in cooperation with the German Agricultural Team of the German Agency of Technical Cooperation (1982).

[46] Zadoks JC, Chang TT, Konzak CF (1974). A decimal code for the growth stages of cereals. Weed Res..

[47] Peterson RF, Campbell AB, Hannah AE (1948). A diagrammatic scale for estimating rust intensity on leaves and stems of cereals. Can. J. Res..

[48] Wilcoxson RD, Skovmand B, Atif AH (1975). Evaluation of wheat cultivars for ability to retard development of stem rust. Ann. Appl. Biol..

[49] Statistical Analysis System (SAS) Institute. *SAS/STAT User’s Guide*. 8 (6th Edition) 112. (SAS Institute, 2002).

[50] Tukey JW (1949). Comparing individual means in the analysis of variance. Biometrics.

[51] Falconer DS, Mackey TFC (1996). Introduction to Quantitative Genetics. 3rd Longman Scientific and Technical.

[52] Addinsoft, XLSTAT statistical and data analysis solution. New York, USA (2021). https://www.xlstat.com.

